# Production of Polyhydroxyalkanoates (PHAs) by *Vibrio alginolyticus* Strains Isolated from Salt Fields

**DOI:** 10.3390/molecules26206283

**Published:** 2021-10-17

**Authors:** Hong-Fei Li, Meng-Ru Wang, Lin-Yue Tian, Zheng-Jun Li

**Affiliations:** Beijing Key Laboratory of Bioprocess, College of Life Science and Technology, Beijing University of Chemical Technology, Beijing 100029, China; 17864305359@163.com (H.-F.L.); wmr1561217@163.com (M.-R.W.); tly3110570@163.com (L.-Y.T.)

**Keywords:** *Vibrio alginolyticus*, poly(3-hydroxybutyrate), poly(3-hydroxybutyrate-*co*-3-hydroxyvalerate), PHB, PHBV

## Abstract

*Vibrio alginolyticus* is a halophilic organism usually found in marine environments. It has attracted attention as an opportunistic pathogen of aquatic animals and humans, but there are very few reports on polyhydroxyalkanoate (PHA) production using *V. alginolyticus* as the host. In this study, two *V. alginolyticus* strains, LHF01 and LHF02, isolated from water samples collected from salt fields were found to produce poly(3-hydroxybutyrate) (PHB) from a variety of sugars and organic acids. Glycerol was the best carbon source and yielded the highest PHB titer in both strains. Further optimization of the NaCl concentration and culture temperature improved the PHB titer from 1.87 to 5.08 g/L in *V. alginolyticus* LHF01. In addition, the use of propionate as a secondary carbon source resulted in the production of poly(3-hydroxybutyrate-*co*-3-hydroxyvalerate) (PHBV). *V. alginolyticus* LHF01 may be a promising host for PHA production using cheap waste glycerol from biodiesel refining.

## 1. Introduction

Plastic pollution is a severe and increasing global problem, affecting almost every marine and freshwater ecosystem in the world. The current concerns about surrounding environmental pollution caused by discarded petrochemical plastics have prompted the promotion of research on biodegradable materials [1]. Biodegradable polymers such as polyhydroxyalkanoate (PHA), polybutylene succinate (PBS), and polylactic acid (PLA) are regarded as major alternatives to traditional petrochemical plastics [2]. PHA is a type of polyester obtained by the esterification and polymerization of hydroxyacyl-CoA monomers [3]. It possesses similar mechanical characteristics to those of traditional petroleum-based plastics, while offering advantages such as biodegradability, biocompatibility, and renewable production [4]. Thus, PHA is considered to be one of the most promising green materials [3,4,5].

Poly(3-hydroxybutyrate) (PHB) is the most common and most well-studied PHA, obtained by the homopolymerization of 3-hydroxybutyrate (3HB) units. Due to its good oxidation resistance, water resistance, and hardness, PHB was mainly developed into packaging materials [6]. Different monomers, such as 3-hydroxyvalerate (3HV), can also be incorporated into the polymer. For example, copolymerizing 3HB and 3HV monomers yielded poly(3-hydroxybutyrate-*co*-3-hydroxyvalerate) (PHBV) [7]. Most bacteria require the external addition of precursors such as propionate or valerate to synthesize PHBV. The introduction of 3HV monomers into the PHBV copolymer improved the overall quality of PHA, while the physical, chemical, and mechanical properties could be modulated by changing the monomer composition of the copolymer [7,8].

In nature, microorganisms accumulate PHA as a way to survive under stressful and unstable environmental conditions [3]. PHA is synthesized by many microorganisms under conditions of an unbalanced nutrient supply, and this has been used to achieve the large-scale fermentation of PHA. However, the commercial production of PHA is far from being able to meet the market demand. Notably, the high production cost of PHA (2.2–5.0 Euro/kg) greatly limits its application as a green material [9]. The production cost of PHA is mainly attributed to the price of fermentation feedstocks and the energy consumption of the sterilization process [3,10]. Based on this, various research teams attempted to reduce the production cost of PHA by increasing the yield of strains, using cheap materials, and developing non-sterile fermentation technology [5,9,11]. For example, waste fish oil and glycerol were employed to produce PHB using a novel halophilic bacterium *Salinivibrio* sp. M318 isolated from fermenting shrimp paste [12]. Another halophile, *Halomonas* TD01, isolated from Aydingol Lake of Xinjiang of China was shown to be a hyper PHA producer, which could grow without contamination under unsterile conditions at high salt/pH conditions [13]. Metabolic engineering and synthetic biology strategies have been applied to obtain superior PHA-producing bacteria capable of rapid growth, high yield, and with the ability to synthesize various types of PHAs [14]. With the engineering of cell-shape-related genes including *mreB*, *ftsA*, and *sulA*, the cell size of *Halomonas* was enlarged to offer more space for PHA synthesis and facilitate cell precipitations for downstream purification [15,16]. Furthermore, the directed evolution of PHA synthase altered enzyme specificity and enabled the polymerization of lactyl-CoA to produce the non-natural lactate-containing polymers, including poly(lactate-*co*-3-hydroxybutyrate) and poly(lactate-*co*-glycolate) [17,18]. Similarly, aromatic polyesters were also synthesized with evolved CoA-transferase and PHA synthase [19].

*Vibrio* strains are widely distributed in seawater and marine animals in estuaries, bays, and coastal areas. Their cells are relatively short, generally curved in a sharp arc or straight rod shape, with slender flagella [20]. For halophilic bacteria, there is limited knowledge on the production of PHA by *Vibrio* strains. Nevertheless, several species of *Vibrio* strains including *V. harveyi* [21,22], *V. natriegens* [23], *V. azureus* [24], and *V. fischeri* [25] were identified for PHA production. Recently, *Vibrio proteolyticus* isolated from the Korean marine environment in an M9 basic medium containing 2% (*w/v*) fructose, 0.3% (*w/v*) yeast extract, and 5% (*w/v*) sodium chloride (NaCl) exhibited a high PHA content (54.7 wt%) and biomass (4.94 g/L) [26]. In addition, comparative genomics of marine bacteria from a plastic biodegradation consortium with the capacity to degrade PHA revealed the presence of PHA-related genes in *V. harveyi*, *V. natriegens*, and *V. proteolyticus*. Interestingly, a series of *Vibrio* species including *V. alginolyticus*, *V. furnissii*, *V. tubiashii*, *V. diabolicus*, *V. rotiferianus*, *V. atypicus*, *V. parahaemolyticus*, and *V. campbellii* were all identified to possess the key enzymes in the PHA biosynthesis pathway [27]. The findings could indicate the PHA accumulation capacity of *Vibrio* strains, yet cultivation studies should be explored to identify their detailed potential for PHA production. 

As salt-tolerant bacteria from the marine environment can survive in high-salt media with low contamination, they do not require certain steps such as antibiotic treatment or sterilization to provide an economical biological process for PHA production [22]. Marine microorganisms may become strong candidates for the production of PHA due to their adaptation to high-salt conditions and the potential to utilize cost-effective seawater as a culture medium [26]. In this study, two strains of *V. alginolyticus* were screened from the Tianjin Saltworks, named *V. alginolyticus* LHF01 and *V. alginolyticus* LHF02, and were found to be capable of fermenting various carbon sources to produce PHB. The accumulation of PHB in the cells of *V. alginolyticus* with glycerol as the carbon source was observed by transmission electron microscopy. After optimizing the NaCl concentration and incubation temperature, the PHB titer of *V. alginolyticus* LHF01 was improved to 5.08 g/L. When 4 g/L of propionate was added, *V. alginolyticus* LHF01 produced 1.44 g/L of P(3HB-*co*-24.42 mol% 3HV).

## 2. Materials and Methods

### 2.1. Sample Collection and Strain Screenings

Soil samples were collected at 2 m intervals at a depth of 0–10 cm from the salt fields near the Central Avenue of Tianjin Binhai, China (117°36′ E, 38°54′ N) on 12 November 2020. Each sample was diluted with autoclaved 0.8% (*w/v*) NaCl solution, and then spread on solid TYS plates containing (g/L) NaCl 20, KCl 0.7, CaCl_2_·2H_2_O 1.4, MgSO_4_·7H_2_O 6.8, MgCl_2_·6H_2_O 5.4, NaHCO_3_ 0.2, yeast extract 1, peptone 5, and 1.5% (*w/v*) agar. The plates were incubated at 30 °C for 2 days, and colonies with different morphological characteristics were separated from each plate. Different colonies were selected and cultured in a liquid TYS medium supplemented with 20 g/L glycerol as a carbon source for 2 days to screen the possible accumulation of PHB. At the same time, cryostock containing 50% glycerol (*w/v*) was prepared and stored at −80 °C for further use.

### 2.2. 16S rDNA Sequencing and Homology Analysis

The variable region of the 16S rDNA was amplified using the universal primers 27F (5′ AGA GTT TGA TCM TGG CTC AG) and 1492R (5′ TAC GGY TAC CTT GTT ACG ACT T). The PCR program encompassed an initial denaturation step at 94 °C for 3 min, followed by 30 cycles of denaturation at 94 °C for 30 s, annealing at 55 °C for 30 s, and extension at 72 °C for 1 min, followed by the final extension at 72 °C for 10 min. The amplified 16S rDNA target amplicon was tested for purity and size by agarose gel electrophoresis, and then ligated into the pEasy-Blunt vector (TransGen Biotech, Beijing, China). Next, the ligation product was electroporated into *E. coli* competent cells and transformants were cultured for 14–16 h. Colony PCR was performed using the primers M13F (5′ TGT AAA ACG ACG GCC AGT) and M13R (5′ CAG GAA ACA GCT ATG ACC) to obtain the complete 16S rDNA. Sequencing was conducted by BGI Group (Shenzhen, China), followed by identification at the species level using EZBiocloud (http://www.ezbiocloud.net, accessed on 13 October 2021) for homology analysis. The phylogenetic tree of 16S rDNA sequences was constructed with Molecular Evolutionary Genetics Analysis (MEGA, version 7.0, https://www.megasoftware.net/, accessed on 13 October 2021) software using the Neighbor-Joining method.

### 2.3. Cell Growth Rate and Antibiotic Resistance Tests of V. alginolyticus

A single colony of the *V. alginolyticus* strain was picked from the TYS plate and transferred into a test tube containing 4 mL of TYS medium, then cultured overnight in a shaker at 37 °C and 200 rpm. An appropriate amount of the resulting seed culture was used to inoculate a 250 mL Erlenmeyer flask containing 30 mL of TYS medium supplemented with 20 g/L glycerol to an initial OD_600_ of 0.1 and cultivated at 37 °C and 200 rpm. The doubling time was calculated in the log phase of cell growth as reported by Eagon [28], using the formula

(1) $$g=t/n\;n=\left({log}_{10}y/x\right)/{log}_{10}2$$

where g is the generation time, t is the detection time interval (h), n is the number of generations, y is the end-point OD_600_, and x is the starting OD_600_. *E. coli* K12(DE3) cultivated at 37 °C and 200 rpm in the Luria–Bertani (LB) medium was included as a control. The LB medium contained 5 g/L yeast extract, 10 g/L Bacto tryptone, and 10 g/L NaCl. 

To determine the antibiotic resistance of *V. alginolyticus*, common antibiotics including ampicillin, kanamycin, spectinomycin, and chloramphenicol were added to TYS plates at a series of antibiotic gradients, then 100 μL overnight culture of *V. alginolyticus* were spread on the TYS plates and incubated at 37 °C for 24 h. The growth of colonies on the TYS plates was observed.

### 2.4. Culture Conditions for PHA Production

To perform PHA production experiments in shake flasks, 20 μL of glycerol stock was transferred into 20 mL of sterile TYS medium and incubated at 37 °C and 200 rpm for 16 h. Next, 1.5 mL of the resulting seed culture was transferred into 30 mL of sterilized TYS medium in a 250 mL Erlenmeyer flask and cultivated at 37 °C and 200 rpm for 24 h. A series of carbon sources were added to the medium to investigate the carbon utilization and PHA production profiles. The substrates include 20 g/L sugars (glucose, sucrose, xylose, soluble starch), 20 g/L glycerol, 10 g/L organic acids (acetate, butyrate, succinate, citrate), and 10 g/L higher fatty acids (myristic acid, palmitic acid, stearic acid, oleic acid, linoleic acid). To optimize the culture conditions for PHA production, different incubation temperatures (25–42 °C) and NaCl concentrations (0–90 g/L) were tested. In addition, propionate at different concentrations (2–6 g/L) was added to the medium as a secondary carbon source to observe the possible production of PHBV copolymer.

### 2.5. Quantification of PHA and Cell Growth 

To determine the dry cell weight (CDW) and intracellular PHA content, 30 mL of the strain culture broth were centrifuged at 10,000 *g* for 10 min, after which the cell pellet was rinsed twice with distilled water, transferred to a pre-weighed test tube and lyophilized. The weight of the empty centrifuge tube and the total weight of the centrifuge tube and the freeze-dried solids were measured to calculate the CDW. About 30–40 mg of lyophilized cells was transferred into the esterification tube, 2 mL of esterification solution (methanol solution with 3% H_2_SO_4_) and 2 mL chloroform was added, followed by incubation in an oven at 100 °C for 4 h. After the esterification was completed, 1 mL of deionized water was added for stratification, and the lower organic phase containing methyl-3-hydroxyalkanoates was used for gas chromatography (GC). The GC was conducted using an HP-5 column (30 m, 0.25 mm) and a flame-ionization detector. PHBV purchased from Sigma-Aldrich (St. Louis, MO, USA) was used as the analytical standard.

### 2.6. Transmission Electron Microscopy (TEM) Analysis of Cell Morphology

To observe the morphological changes related to cell growth on glycerol as the carbon source, *V. alginolyticus* strains LHF01 and LHF02 were cultured in TYS medium supplemented with 20 g/L glycerol for 24 h for TEM observation. The strains cultivated in TYS medium without an additional carbon source were used as the control group. The cell culture was centrifuged to collect the bacterial pellet, washed 1–2 times with PBS and mixed with a pre-cooled fixative solution, and stored at 4 °C for at least 12 h. The fixative solution was poured out, then the samples were rinsed 3 times with a 0.1 M, pH7.0 phosphate buffer. Then, the samples were fixed with a 1% osmium acid solution for 2 h, rinsed 3 times with 0.1 M, pH7.0 phosphate buffer, and dehydrated with graded ethanol solutions and acetone. The samples were then embedded in a mixture of embedding agent and acetone, sliced using a Leica Ultracut S ultramicrotome, and stained with a lead citrate solution and a uranyl acetate 50% ethanol saturated solution. The prepared sample was visualized using a JEM-2100F transmission electron microscope (JEOL Ltd., Tokyo, Japan). 

### 2.7. PHA Extraction and Molecular Weight Determination

The dried cell pellets were transferred to a screw-capped tube and mixed with chloroform (10%, *w/v*). The tube was sealed off and maintained at 100 °C for 4 h, then mixed with deionized water and centrifuged to obtain the organic phase. The chloroform phase was collected and PHA was precipitated by the addition of a 10-fold volume of ice-cold ethanol. Gel permeation chromatography (GPC) was performed to measure the number-average molecular weight (*Mn*), weight-average molecular weight (*Mw*), and polydispersity of PHA. The purified polymer sample was dissolved in chloroform at a concentration of 3 mg/mL, filtered, and subjected to analysis using GPC (LC-20AD, Shimadzu, Kyoto, Japan) equipped with Shodex K-804 column (Waters, Milford, MA, USA). Chloroform was used as the eluent at a flow rate of l ml/min. Polystyrene standards purchased from Sigma-Aldrich (St. Louis, MO, USA) were used for calibration.

## 3. Results and Discussion

### 3.1. Isolation of V. alginolyticus Strains

**Figure 1:**
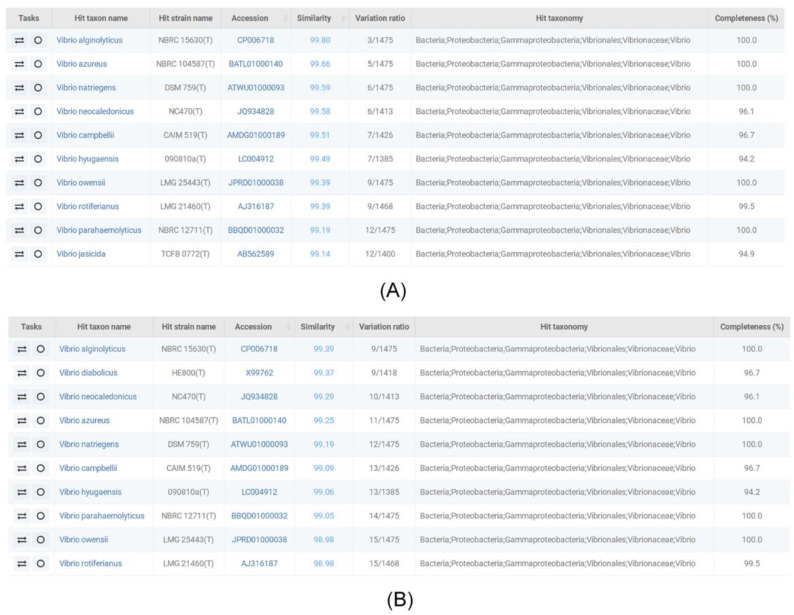
Homology analysis of the 16S rDNA sequences of LHF01 (**A**) and LHF02 (**B**).

The collected soil samples were cultured in TYS medium supplemented with glycerol, and 50 colonies were screened for the possible accumulation of PHA polymers. The two isolates LHF01 and LHF02 were found to produce PHB through GC analysis. Subsequently, the two strains were identified by 16S rDNA sequencing. The sequencing results were analyzed for homology using the taxonomically united database of 16S rRNA gene sequences EzBioCloud [29], and the first ten homologs are shown in Figure 1. The results showed that the 16S rDNA sequences of LHF01 and LHF02 were most identical to that of *V. alginolyticus* NBRC15630. The phylogenetic analysis using MEGA7.0 software also showed their closest match with *V.alginolyticus* strain NBRC15630 (Supplementary Figure S1).

**Figure 2:**
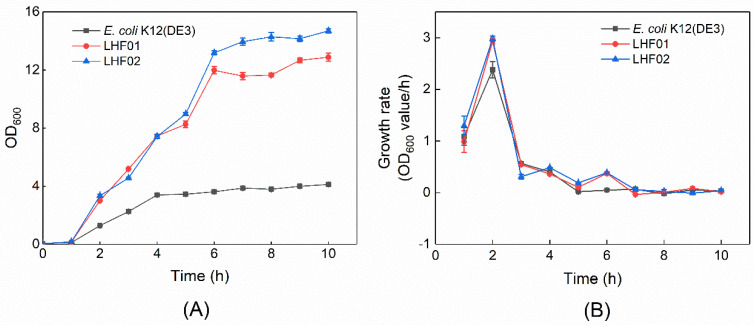
The cell growth curve (**A**) and growth rate (**B**) of *V. alginolyticus* and *E. coli*. *V. alginolyticus* strains were cultivated in TYS medium supplemented with 20 g/L glycerol, while *E. coli* K12 (DE3) was cultivated in LB medium. Data are expressed as averages and standard deviations of three parallel experiments. The growth rate was calculated using OD_600_ values according to the following equation: μ = (lnX2 − lnX1)/(t2 − t1). X1 and X2 refer to the OD_600_ values of cell cultures at culture time (t) t1 and t2.

*V. alginolyticus* LHF01 and LHF02 were then cultivated in TYS medium supplemented with a series of different antibiotics to study their drug resistance. Both strains exhibited complete resistance to ampicillin and kanamycin, weak resistance to spectinomycin, and sensitivity to chloramphenicol (Table 1). In addition, both strains exhibited rapid cell growth in TYS medium supplemented with glycerol during the screening experiment for PHA production. Detailed cell growth curves of *V. alginolyticus* LHF01 and LHF02 were recorded, and the model bacterium *E. coli* K12 (DE3) was included as a control (Figure 2). The cell growth rates of *V. alginolyticus* LHF01 and LHF02 were both higher than that of *E. coli* K12 (DE3). The OD_600_ of LHF01 and LHF02 reached over 12 after 6 h of cultivation, while the maximal OD_600_ of *E. coli* was only about 3. The doubling time of *V. alginolyticus* LHF01 and LHF02 in TYS medium containing glycerol was calculated to be 14.16 and 13.95 min, respectively, while the doubling time of *E. coli* K12 (DE3) in LB liquid medium was 17.53 min. The fast-growth characteristics of *V. alginolyticus* would be an advantage in the fermentation industry.

### 3.2. PHB Production by V. alginolyticus LHF01 and LHF02 on Various Substrates

**Figure 3:**
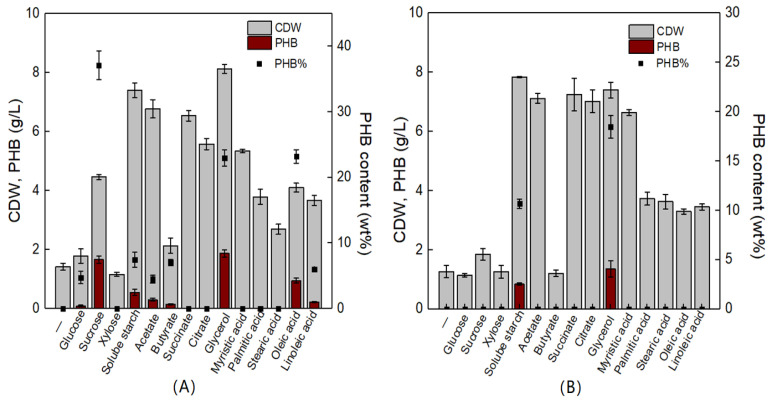
CDW, PHB concentration, and PHB content profiles of *V. alginolyticus* LHF01 (**A**) and LHF02 (**B**) cultivated in TYS medium with different carbon sources. Data are expressed as averages and standard deviations of three parallel experiments.

*V. alginolyticus* is reported to be a halophilic organism that is widely distributed in marine environments. Together with *V. harveyi* and *V. parahaemolyticus, V. alginolyticus* is one of the three dominant *Vibrio* species in the ocean. It is also an important pathogen that can infect a variety of fish, shrimp, and shellfish, causing significant losses to the aquaculture industry around the world [30]. Interestingly, we isolated two strains of *V. alginolyticus,* which were capable of producing PHB. Next, the effects of various carbon sources on the cell growth and PHB biosynthesis of *V. alginolyticus* LHF01 and LHF02 were studied. The results showed that *V. alginolyticus* LHF01 could synthesize PHB from a series of carbon sources including glucose, sucrose, soluble starch, acetate, butyrate, glycerol, oleic acid, and linoleic acid, while *V. alginolyticus* LHF02 produced PHB only using soluble starch and glycerol (Figure 3). For *V. alginolyticus* LHF01, sucrose, glycerol, and oleic acid yielded PHB contents of 37.14%, 22.96%, and 23.21%, respectively. A maximal PHB titer of 1.87 g/L was obtained using glycerol as the carbon source. In terms of *V. alginolyticus* LHF02, glycerol was also the most favorable substrate for PHB accumulation, reaching a titer of 1.36 g/L. Although acetate, succinate, citrate, and myristic acid supported effective cell growth in *V. alginolyticus* LHF02 and the CDW reached ~7 g/L, no PHB accumulation was observed with any of these carbon sources. The results indicated that *V. alginolyticus* LHF01 is more suitable for PHB production than *V. alginolyticus* LHF02. To achieve low-cost PHA production, it is crucial to find economically feasible substrates and efficient hosts. The utilization of crude glycerol from biodiesel production by *V. alginolyticus* strains would help reduce the production cost and increase the market competitiveness of PHA.

**Figure 4:**
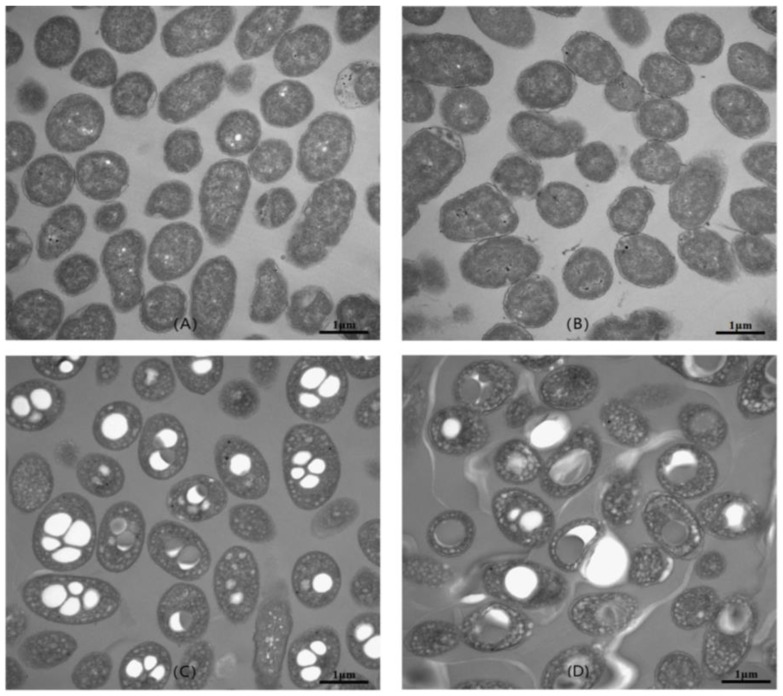
TEM micrographs of *V. alginolyticus* LHF01 (**A**,**C**) and LHF02 (**B**,**D**). Strains were cultivated in TYS medium (**A**,**B**) or TYS medium supplemented with 20 g/L glycerol (**C**,**D**) at 37 °C and 200 rpm for 24 h.

The cells of *V. alginolyticus* LHF01 and LHF02 grown on TYS medium and TYS supplemented with glycerol were observed by transmission electron microscopy. As shown in Figure 4, the average cell size of *V. alginolyticus* cultured in TYS medium was found to be 0.8–1.8 μm. With the addition of 20 g/L glycerol, it was observed that the volume of the cells increased significantly, and the cells contained electron-translucent inclusions of different sizes. The TEM observation results were consistent with previous studies, in which the intracellular accumulation of PHA polymer resulted in visible particles [13].

Next, the polymers produced by *V. alginolyticus* LHF01 and LHF02 grown on TYS medium supplemented with 20 g/L glycerol were extracted and subjected to molecular weight analysis. The molecular weight of PHA synthesized by microorganisms usually ranges from 200 to 30,000 kDa [31]. As shown in Table 2, the weight-average molecular weight of PHB produced by *V. alginolyticus* LHF01 was 201 kDa, while *V. alginolyticus* LHF02 produced PHB with a weight-average molecular weight of 1380 kDa. The *Mw* was similar to those of PHA polymers produced by other halophilic bacteria such as *Salinivibrio* sp. M318 [12], *Salinivibrio* sp. TGB10 [31], *Halomonas sp.* O-1, and *H. elongata* DSM2581 [32]. The polydispersity (*Mw*/*Mn*) of the polymer produced by *V. alginolyticus* LHF02 was much higher than that of *V. alginolyticus* LHF01. Generally, the mechanical strength of PHA is positively correlated with the molecular weight, thus polymers with high Mw would be preferred. However, the precise mechanism of PHA molecular weight regulation is still unclear [33]. PHA synthase, which catalyzes the polymerization of acyl-CoA monomers, is considered the critical factor determining the molecular weight of PHA. The presence of a chain transfer agent and simultaneous degradation of PHA also contributed to molecular weight regulation [34]. The differences in PHA molecular weights between *V. alginolyticus* LHF01 and LHF02 were probably due to the intracellular expression and catalytic activity of their PHA synthases, which requires in-depth research in the future.

### 3.3. Optimization of Culture Conditions for PHB Production

**Figure 5:**
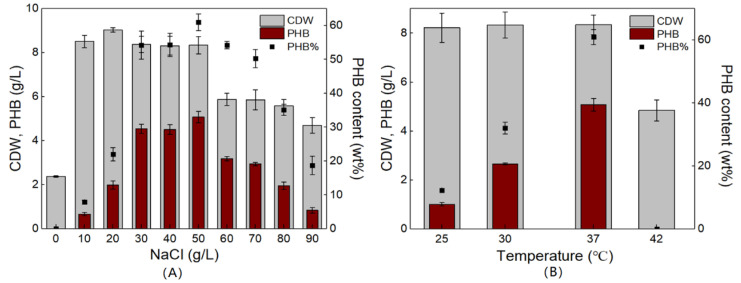
CDW, PHB concentration, and PHB content profiles of *V. alginolyticus* LHF01 cultivated in TYS medium supplemented with 20 g/L glycerol at different NaCl concentrations (**A**) and temperatures (**B**). Data are expressed as averages and standard deviations of three parallel experiments.

In experiments with different carbon sources, *V. alginolyticus* LHF01 produced more PHB than LHF02 when glycerol was used as the carbon source. Next, the culture conditions were optimized with the aim of increasing PHB production from glycerol using *V. alginolyticus* LHF01. Since *V. alginolyticus* is a halophilic bacterium, TYS media containing different concentrations of NaCl (0–90 g/L) were tested first. As shown in Figure 5A, the cell growth was extremely poor without the NaCl addition and no PHB accumulation was observed, indicating that NaCl is essential for efficient cell growth and PHB accumulation by *V. alginolyticus*. When the NaCl concentration was increased to 30–50 g/L, the CDW of *V. alginolyticus* LHF01 exceeded 8 g/L, and the PHB content in the cells was over 50%. The highest PHB titer of 5.08 g/L was obtained at a NaCl concentration of 50 g/L. When the concentration of NaCl was increased above 50 g/L, PHB accumulation gradually decreased and only 0.84 g/L PHB was produced at 90 g/L NaCl.

Temperature optimization is important for microorganisms used for PHA production. For example, the *Vibrio* strain BTKB33 accumulated the maximal amount of PHA at a temperature of 30–35 °C [24]. To optimize the temperature, *V. alginolyticus* LHF01 was cultivated at 25 °C, 30 °C, 37 °C, and 42 °C with 20 g/L glycerol and 50 g/L NaCl to determine the suitable temperature for PHB production. *V. alginolyticus* LHF01 produced the highest PHB titer at 37 °C. It is usually observed that CDW is directly related to the amount of PHA produced. Interestingly, *V. alginolyticus* LHF01 maintained CDW of about 8 g/L, but the PHB production decreased when the cultivation temperature decreased to 25 °C (Figure 5B). We speculated that the marine bacterium *V. alginolyticus* LHF01 can survive at a wide range of cultivation temperatures, but low temperatures may decrease the catalytic activity of PHA synthesis enzymes. When the cultivation temperature was increased to 42 °C, *V. alginolyticus* LHF01 showed poor cell growth and no PHB production was observed, indicating that the strain is not suitable for growth at high temperatures.

### 3.4. PHBV Production by V. alginolyticus

The copolymer of 3-hydroxybutyrate and 3-hydroxyvalerate, named PHBV, has been produced on the industrial scale by Ningbo Tianan Co. for many years. The incorporation of a 3HV monomer alters the properties of polyester, making the PHBV more flexible and tougher than the homopolymer of 3-hydroxybutyrate (PHB) [35]. Propionate is the most common secondary carbon source used to generate the 3HV monomer for PHBV synthesis [3]. Therefore, glycerol and propionate were simultaneously provided as carbon sources to investigate the cell growth and possible production of PHBV by *V. alginolyticus* LHF01. The NaCl concentration and culture temperature were kept at 50 g/L and 37 °C, respectively. The glycerol concentration of 20 g/L and propionate concentration was varied at 2, 4, and 6 g/L. As shown in Table 3, increasing the propionate concentration to 4 g/L led to the accumulation of 1.44 g/L PHBV copolymer, with a 3HV monomer content of 24.42 mol%. However, a further increase in the propionate concentration to 6 g/L severely inhibited cell growth and no PHA accumulation was detected. Previously, the use of fructose and propionate as co-substrates to cultivate *V. proteolyticus* enabled the accumulation of the PHBV copolymer containing 15.8% 3HV, but a propionate concentration higher than 3 g/L inhibited cell growth [26]. Thus, *V. alginolyticus* LHF01 showed better propionate tolerance than *V. proteolyticus,* and the PHA synthase possesses broad substrate specificity.

The genus *Vibrio* is widely distributed in nature, especially in aqueous environments. *Vibrio* species usually require salt and can grow over a wide temperature range [20]. The major pathogens from this genus include *V. cholerae*, *V. parahaemolyticus*, and *V. vulnificus*, which are capable of causing disease in humans and domestic animals [36]. However, there are very few reports on PHA production using *Vibrio* species (Table 4). The identified *Vibrio* PHA producers include *V. harveyi*, *V. fischeri*, *V. proteolyticus*, *V. azureus*, *V. natriegens*, and several strains that have not been assigned to specific species [21,22,23,24,25,26,37]. Here, we report that the *V. alginolyticus* strain LHF01 can effectively produce PHB using glycerol as the carbon source. Our study also achieved the highest PHB titer in *Vibrio* species. Glycerol is the major byproduct of the biodiesel industry and may be employed as a cost-effective substrate for PHA production. *V. alginolyticus* showed favorable cell growth and PHB accumulation in the media containing 50 g/L NaCl, which could be a suitable concentration to inhibit the growth of common bacteria that lack salt resistance [38]. Currently, the PHB titer obtained from *V. alginolyticus* was lower than those produced by other halophiles such as the haloarchaeon *Haloferax mediterranei* and *Halomonas* [11]. Considering that *Vibrio* species such as *V. natriegens* and *V. alginolyticus* are fast-growing hosts with reported doubling times of less than 10 min, they may be developed into superior next-generation microbial chassis for biotechnological applications [39]. Further development of genetic manipulation tools to engineer the carbon degradation and PHA synthesis pathways of *V. alginolyticus* may yield a powerful new chassis for the hyper-production of PHA.

## 4. Conclusions

In this study, we isolated the two *V. alginolyticus* strains LHF01 and LHF02 with the ability to produce PHA. Strain LHF01 accumulated PHB when grown on sucrose, soluble starch, acetate, butyrate, glycerol, oleic acid, and linoleic acid, among which glycerol yielded the highest PHB titer of 1.87 g/L. Compared with cells cultured in a medium without glycerol, the size of the cells was significantly increased, and there were many PHA particles in the cells. Further optimization of culture conditions increased the PHB titer to 5.08 g/L. When 4 g/L of propionate was provided as a secondary carbon source, LHF01 produced 1.44 g/L of P(3HB-*co*-24.42 mol% 3HV). *Vibrio* species such as *V. natriegens* and *V. alginolyticus* are fast-growing hosts, thus further development of genetic tools and metabolic engineering of *V. alginolyticus* would help to construct a novel chassis for effective PHA fermentation.

## Figures and Tables

**Table 1:** Antibiotic resistance of *V. alginolyticus* LHF01 and LHF02.

	Antibiotics (μg/mL)	10	25	50	80	100	150
*V. alginolyticus* LHF01	Chloramphenicol	-	-	-	-	-	-
	Ampicillin	++	++	++	++	++	++
	Kanamycin	++	++	++	+	+	-
	Spectinomycin	++	+	+	-	-	-
*V. alginolyticus* LHF02	Chloramphenicol	+	-	-	-	-	-
	Ampicillin	++	++	++	++	++	++
	Kanamycin	++	++	+	+	-	-
	Spectinomycin	++	+	+	-	-	-

++: The plate was covered with a bacterial lawn. +: There were individual colonies growing on the plate, but no lawn was formed. -: There was no colony growth.

**Table 2:** Molecular weight of PHB produced by *V. alginolyticus* LHF01 and LHF02.

Strain	Carbon Source	Mw (×10^3^ Da)	Mn (×10^3^ Da)	Mw/Mn
*V. alginolyticus* LHF01	glycerol	201	90	2.2
*V. alginolyticus* LHF02	glycerol	1380	323	4.3

Mw, weight-average molecular weight; Mn, number-average molecular weight.

**Table 3:** Production of PHBV by *V. alginolyticus* LHF01 using glycerol and propionate.

Carbon Source	CDW (g/L)	PHA Content (wt%)	PHA (g/L)	3HB (mol%)	3HV (mol%)
Gly + PA(2) *	8.47 ± 0.32	13.48 ± 0.35	1.14 ± 0.02	100	ND
Gly + PA(4)	8.55 ± 0.43	16.61 ± 0.49	1.44 ± 0.16	75.58 ± 0.25	24.42 ± 0.25
Gly + PA(6)	1.83 ± 0.06	ND	ND	ND	ND

Shake-flask fermentations were performed at 37 °C and 200 rpm with 20 g/L of glycerol and propionate for 24 h. Propionate was applied at different concentrations (2, 4, and 6 g/L). Data are expressed as the averages and standard deviations of three parallel experiments. Gly, glycerol; PA, propionate; CDW, cell dry weight. * Propionate concentration, indicated in g/L. ND, not detected.

**Table 4:** PHA production by *Vibrio* strains.

Microorganism	Carbon Source	PHAs	CDW (g/L)	PHA Content (wt%)	PHA Titer (g/L)	Reference
*V. harveyi*	Glycerol	PHB	(OD_660_) 4.2	2.6	—	[21]
*V. harveyi* *MCCB 284*	Glycerol	PHB	3.2	72	2.3	[22]
*Vibrio* sp. M11	Glycerol	PHB	(OD_600_) 12.2	30.2	—	[23]
*V. azureus* BTKB33	Glucose	PHB	1.12	42.69	0.48	[24]
*V. fischeri* 1231	Glycerol	PHB	—	0.4	0.003	[25]
*V. proteolyticus*	Fructose	PHB	4.94	54.7	2.7	[26]
*V. proteolyticus*	Fructose+ Propionate	P(3HB-*co*-15.8% 3HV)	—	—	—	[26]
*Vibrio* sp. KN01	Soybean oil	PHB	2.4	8	0.19	[37]
*Vibrio* sp. KN01	Soybean oil	P(3HB-*co*-14%3HP-*co*-3%5HV)	0.4	40	0.16	[37]
*V. alginolyticus* LHF01	Glycerol	PHB	8.34	60.97	5.08	This study
*V. alginolyticus* LHF01	Glycerol+ Propionate	P(3HB-*co*-24.42% 3HV)	8.55	16.11	1.44	This study

## Data Availability

The data presented in this study are available in the article.

## Supplementary Materials

The following are available online. Figure S1: The Neighbor-joining tree based on 16S rDNA gene sequences showing the phylogenetic relationships of *V. alginolyticus* LHF01/LHF02 and some other related taxa.

## Sample Availability

Samples of the bacteria are available from the authors.
