# Supplementary material for: Production of Polyhydroxyalkanoates (PHAs) by Vibrio alginolyticus Strains Isolated from Salt Fields
Source: Molecules. 2021 Oct 17;26(20):6283. doi: 10.3390/molecules26206283 (PMC8537743; doi:10.3390/molecules26206283)

Supplementary materials

Figure S1. The Neighbor-joining tree based on 16S rDNA gene sequences showing the phylogenetic relationships of *V. alginolyticus* LHF01/LHF02 and some other related taxa. Bootstrap values indicated at nodes.

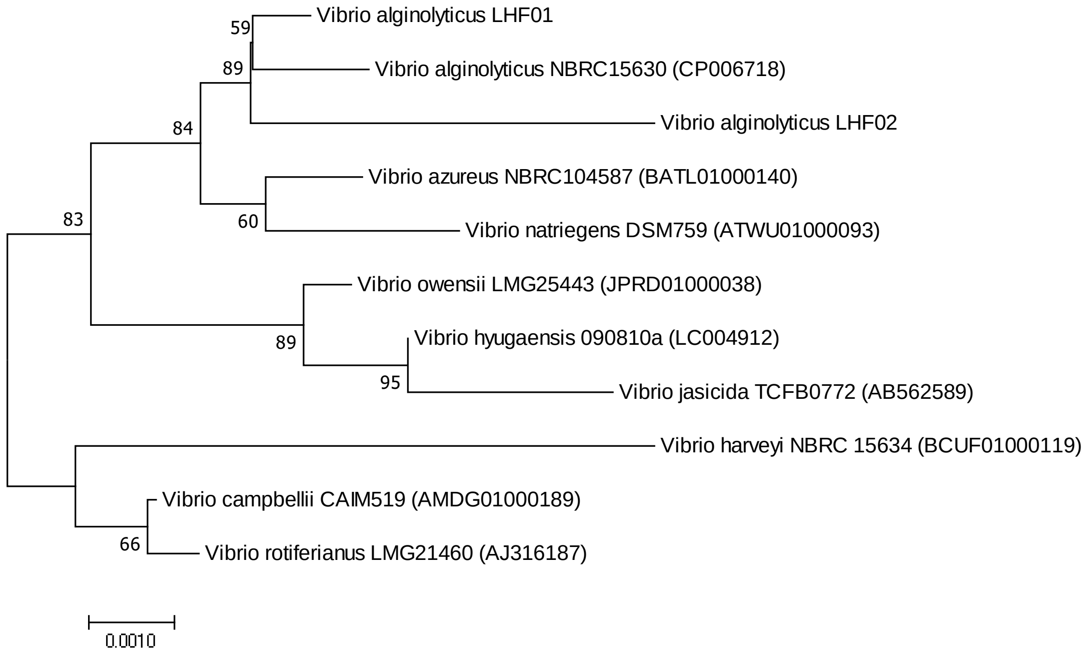

Supplement: Supplementary file 1 [file molecules-26-06283-s001.zip › molecules-1393821-Supplementary.pdf]
